# Classifying equivalence relations in the Ershov hierarchy

**DOI:** 10.1007/s00153-020-00710-1

**Published:** 2020-02-13

**Authors:** Nikolay Bazhenov, Manat Mustafa, Luca San Mauro, Andrea Sorbi, Mars Yamaleev

**Affiliations:** 1 Sobolev Institute of Mathematics, pr. Akad. Koptyuga 4, Novosibirsk, Russia 630090; 2 Novosibirsk State University, ul. Pirogova 2, Novosibirsk, Russia 630090; 3 Department of Mathematics, School of Science and Technology, Nazarbayev University, 53, Kabanbay Batyr Avenue, Astana, 010000 Republic of Kazakhstan; 4 Institute of Discrete Mathematics and Geometry, Vienna University of Technology, Vienna, Austria; 5 Dipartimento di Ingegneria Informatica e Scienze Matematiche, Università Degli Studi di Siena, 53100 Siena, Italy; 6 Kazan Federal University, 18 Kremlyovskaya str., Kazan, Russia 420008

**Keywords:** Computability theory, Ershov hierarchy, $\Delta ^0_2$ equivalence relations, Computably enumerable equivalence relations, 03D55

## Abstract

Computably enumerable equivalence relations (ceers) received a lot of attention in the literature. The standard tool to classify ceers is provided by the computable reducibility $\leqslant _c$. This gives rise to a rich degree structure. In this paper, we lift the study of *c*-degrees to the $\Delta ^0_2$ case. In doing so, we rely on the Ershov hierarchy. For any notation *a* for a non-zero computable ordinal, we prove several algebraic properties of the degree structure induced by $\leqslant _c$ on the $\Sigma ^{-1}_{a}\smallsetminus \Pi ^{-1}_a$ equivalence relations. A special focus of our work is on the (non)existence of infima and suprema of *c*-degrees.

## Introduction

Computable reducibility is a longstanding notion that allows classifying equivalence relations on natural numbers according to their complexity.

### Definition 1.1

Let *R*, *S* be equivalence relations with domain $\omega$. *R* is *computably reducible* to *S*, denoted $R \leqslant _c S$, if there is a computable function *f* such that, for all $x,y \in \omega$,

We write $f: R \leqslant _c S$ to denote that *f* is a computable function that reduces *R* to *S*; *c*-degrees are introduced in the standard way.

The history of computable reducibility has many roots, being often rediscovered and explored in connection with different fields. Its study dates back to the fundamental work of Ershov in the theory of numberings, where the reducibility is introduced in a category-theoretic fashion (see Ershov’s monograph [11] in Russian, or [12] for an English survey). In the 1980s, computable reducibility proved to be a fruitful tool for calibrating the complexity of provable equivalence of formal systems and scholars focused mostly on the $\Sigma ^0_1$ case (see, e.g., [5, 19, 26]). Following Gao and Gerdes [17], we adopt the acronym “ceers” to refer to computably enumerable equivalence relations. The interested reader can consult Andrews et al. [1] for a nice and up-to-date survey on ceers, with a special focus on *universal ceers*, i.e., ceers to which all other ceers are computably reducible. The degree of universal ceers is by now significantly explored: for instance, in [2] the authors proved that all uniformly effectively inseparable ceers are universal. A complementary line of research aims at providing concrete examples of universal ceers. To this end, Nies and Sorbi [21] constructed a finitely presented group whose word problem is a universal ceer.

Far from being limited to ceers, computable reducibility has been also applied to equivalence relations of much higher complexity. Fokina et al. [15] showed that all $\Sigma ^1_1$ equivalence relations are computably reducible to the isomorphism relations on several classes of computable structures (e.g., graphs, trees, torsion Abelian groups, fields of characteristic 0 or *p*, linear orderings). This study was fueled by the observation that computable reducibility represents a nice effective counterpart of *Borel reducibility*, i.e., a key notion of modern descriptive set theory (see [16]). The analogy between Borel and computable reducibility has been explored, for instance, by Coskey et al. [7], who investigated equivalence relations on c.e. sets mirroring classical combinatorial equivalence relations of fundamental importance for Borel theory.

Additional motivation for dealing with computable reducibility comes from the study of c.e. presentations of structures, as is shown for instance in [13, 18] (for a nice survey about c.e. structures, see [23]).

The goal of the present paper is to contribute to this vast (yet somehow unsystematic) research program by making use of computable reducibility to initiate a throughout classification of the complexity of $\Delta ^0_2$ equivalence relations. In this endeavour, we follow and extend the work of Andrews and Sorbi [3], that provides a very extensive analysis of the degree structure induced by computable reducibility on ceers. Ng and Yu [20] broadened the perspective by discussing some structural aspects of the *c*-degrees of *n*-c.e., $\omega$-c.e., and $\Pi ^0_1$-equivalence relations. We similarly rely on the Ershov hierarchy to pursue our analysis.

Although our motivation is rather abstract (and to some extent corresponds to the desire of exporting the guiding questions of classical degree theory to the case of equivalence relations), our object of study shall not be regarded as too much artificial. The following example might convince the reader that $\Delta ^0_2$ equivalence relations occur quite naturally.

Consider the following $\Pi ^0_2$ equivalence relation *R*:Then one can define a “bounded” version of *R*:It is not hard to show that the relation $R^b$ is $\omega$-c.e. Furthermore, the relation $R^b$ admits an interpretation via algebraic structures: One can interpret a number $\langle i,s\rangle$ as the index of a finite linear ordering. Indeed, define the ordering ${\mathcal {L}}_{ i,s}$ as follows. The domain of ${\mathcal {L}}_{i,s}$ is equal to $W_{i}\cap \{ 0,1,\dots ,s\}$, and the ordering on the domain is induced by the standard ordering of natural numbers. Notice that here we assume that ${\mathcal {L}}_{i,s}$ may be empty. The list $({\mathcal {L}}_{i,s})_{i,s\in \omega }$ gives an enumeration of all finite linear orderings, up to permutations of the domains. It is easy to see thatthus the relation $R^b$ can be treated as (one of the possible formalizations of) the relation of isomorphism on the class of finite linear orderings.

### Organization of the paper

In Sect. 2, we set up the stage by offering some disanalogies between the degree structure of ceers and that of $\Delta ^0_2$ equivalence relations. We also prove that infinitely many levels of the Ershov hierarchy contain minimal *c*-degrees. In Sect. 3, we focus on dark degrees, i.e., *c*-degrees not being above the identity on $\omega$: we show that all levels of the Ershov hierarchy, with the exception of $\Pi ^{-1}_1$, contain dark equivalence relations. Sections 4 and 5 are devoted to the existence of infima and suprema of *c*-degrees of $\Delta ^0_2$-equivalence relations: we introduce the notion of mutual darkness and prove that, if $R,S \in \Sigma ^{-1}_a\smallsetminus \Pi ^{-1}_a$ are mutually dark, then *R*, *S* have no infimum in $\Sigma ^{-1}_a\smallsetminus \Pi ^{-1}_a$ and no supremum in $\Delta ^0_2$. It follows that none of the degree structures considered in this paper is neither upper- or a lower-semilattice.

### Notation and terminology

All our equivalence relations have domain $\omega$. Given a number *x*, we denote by $[x]_R$ its *R*-equivalence class. We say that *R* is *infinite* if *R* has infinitely many equivalence classes (otherwise, it is of course *finite*). The following basic equivalence relations will appear many times:

- ${{\,\mathrm{Id}\,}}_n$ is the computable equivalence relation consisting of *n* equivalence classes, i.e.  for all $x,y\in \omega$.
- ${{\,\mathrm{Id}\,}}$ is the identity on $\omega$, i.e.,  if and only if $x=y$.

The following definition is due to Gao and Gerdes [17] (but analogous ways of coding sets of numbers by equivalence relations occur frequently in the literature, see, for instance, the definition of a *set-induced* *c*-degree in [20]).

#### Definition 1.2

An equivalence relation *R* is *n-dimensional* if there are pairwise disjoint sets $A_0,\ldots ,A_{n-1}\subseteq \omega$ such that

$$\begin{aligned} x Ry \Leftrightarrow x=y \vee (\exists i)(x,y \in A_i). \end{aligned}$$

We denote such *R* by $R_{A_0,\ldots ,A_{n-1}}$.

An equivalence relation *R* is *essentially* *n-dimensional* if it has exactly *n* noncomputable equivalence classes.

The following definition comes from [3].

#### Definition 1.3

An equivalence relation *R* is *light* if ${{\,\mathrm{Id}\,}}\leqslant _c R$; it is *dark* if it is not light and has infinitely many equivalence classes.

It is often convenient to think of a given light equivalence relation *R* in terms of some computable listing of pairwise nonequivalent numbers witnessing its lightness. More formally, a *transversal* of an equivalence relation *R* with infinitely many *R*-classes is an infinite set *A* such that $[x]_R\ne [y]_R$, for all distinct $x,y \in A$. (The notion of a transversal, due to [17], has been widely exploited in [3].) It is immediate to see that *R* is light if and only it has a c.e. transversal.

Our computability theoretic notions are standard, see for instance [25]. The basic notions regarding the Ershov hierarchy can be found in [8–10], see also [4]: in particular, recall the following.

#### Definition 1.4

Let *a* be a notation for a computable ordinal. A set $A \subseteq \omega$ of numbers is said to be $\Sigma ^{-1}_a$ (or $A \in \Sigma ^{-1}_a$) if there are computable functions *f*(*z*, *t*) and $\gamma (z,t)$ such that, for all *z* and *t*,

1. $$A(z)=\lim _t f(z, t)$$, with $$f(z,0)=0$$ and $${{\,\mathrm{range}\,}}(f)\subseteq \lbrace 0,1 \rbrace $$;
2. $$\gamma (z,0) =a$$, and
  - $$\gamma (z,t+1)\leqslant _{\mathcal {O}} \gamma (z,t)\leqslant _{\mathcal {O}} a$$;
  - $$f(z,t+1) \ne f(z,t) \Rightarrow \gamma (z,t+1) \ne \gamma (z,t)$$.

We call the function $\gamma$ the *mind-change function* for *A*, relatively to *f*.

A $\Sigma ^{-1}_a$*-approximation pair* to a $\Sigma ^{-1}_a$ set *A*, is a pair $\langle f, \gamma \rangle$, where *f* and $\gamma$ are computable functions satisfying (1), (2) above, for *A*. As is known (see, e.g., [22, 24] for more details), one can give an effective list of $\Sigma ^{-1}_a$-approximation pairs $\langle f_e, \gamma _e \rangle$, so that if $E_e$ is the set whose characteristic function is $\lim _s f_e(\_,s)$, then $(E_e)_{e \in \omega }$ is an effective list of all $\Sigma ^{-1}_a$ sets. Clearly, we can also assume that for every *e* and *s* there is at most one *x* such that $f_e(x,s+1) \ne f_e(x,s)$. Note that we impose this assumption only to the canonical enumeration of the sets lying at any given level of the Ershov hierarchy, and *not* to the equivalence relations that we will construct. So, we can also assume that for every *e* the following is true:

(*) for every *s* there are infinitely (in fact cofinitely) many *x* such that $\gamma _e(x,s)=a$.

Thus we can refer to some effective listing $(E_e)_{e \in \omega }$ of the $\Sigma ^{-1}_a$ sets, where $E_e$ is the set of which $\langle f_e, \gamma _e \rangle$ is a $\Sigma ^{-1}_a$-approximation pair, satisfying (*) as well.

Dually, we say that a set *A* is $\Pi ^{-1}_a$, if ${\overline{A}} \in \Sigma ^{-1}_a$, or equivalently there is a $\Pi ^{-1}_a$*-approximation pair* $\langle f, \gamma \rangle$, i.e. a pair as above but starting with $f(z,0)=1$. We can refer to some effective listing $(E_e)_{e \in \omega }$ of the $\Pi ^{-1}_a$ sets, where $E_e$ is the set of which $\langle f_e, \gamma _e \rangle$ is a $\Pi ^{-1}_a$-approximation pair, satisfying (*) as well.

If ${\mathcal {X}} \in \{\Sigma ^{-1}_a: a \in {\mathcal {O}}\}\cup \{\Pi ^{-1}_a: a \in {\mathcal {O}}\}$ let us call ${\mathcal {X}}^d=\{{\overline{A}}: A \in {\mathcal {X}}\}$ the *dual class* of ${\mathcal {X}}$.

We say that a set *A* is *properly* ${\mathcal {X}}$ if $A \in {\mathcal {X}} \smallsetminus {\mathcal {X}}^d$.

Since any finite ordinal has only one notation, one usually writes $\Sigma ^{-1}_n$ instead of $\Sigma ^{-1}_a$, if *a* is the notation of $n \in \omega$. In analogy with the terminology used for sets, we say that *R* is a *n*-ceer if *R* as a set of pairs is $\Sigma ^{-1}_n$.

## A first comparison with ceers

At first sight, one might expect that the structural properties of the *c*-degrees of ceers are reflected smoothly on the upper levels of the Ershov hierarchy. In this section we show that the parallel is much more delicate.

### Equivalence relations with finitely many classes

Recall that any ceer with finitely many classes is computable. Surely, this is not the case for relations in the Ershov hierarchy. Denote by $F_X$ the equivalence relation with exactly two classes: *X* and ${\overline{X}}$. It is easy to check that $F_X$ is a noncomputable $\Pi ^{-1}_{2n}$ relation if $n>0$ and $X\in \Sigma ^{-1}_n\smallsetminus \Pi ^{-1}_n$. Relations of the form $F_X$ already allow us to demonstrate some simple differences concerning elementary theories of the degree structures:

#### Proposition 2.1

Suppose that ${\mathcal {X}} \in \{ \Pi ^{-1}_a \,:|a|_{{\mathcal {O}}} \geqslant 2\} \cup \{ \Sigma ^{-1}_{a}\,:|a|_{{\mathcal {O}}}\geqslant 3\}$. Then the structure of ${\mathcal {X}}$-equivalence relations is elementarily equivalent to neither ceers nor co-ceers.

#### Proof

Fix a c.e. set *W* such that $\deg _m(W)$ is a minimal *m*-degree. Note that ${{\,\mathrm{Id}\,}}_2 <_c F_W$. Suppose that *E* is an equivalence relation such that ${{\,\mathrm{Id}\,}}_2\leqslant _c E\leqslant _c F_W$. Then *E* is equal to $F_{V}$ for some c.e. set *V* and the minimality of $\deg _m(W)$ implies that either *V* is computable or $V\equiv _m W$. Thus, we have $E\equiv _c {{\,\mathrm{Id}\,}}_2$ or $E\equiv _c F_{W}$.

Hence, the desired elementary difference can be witnessed by the following argument:

1. The (*c*-degree of the) relation ${{\,\mathrm{Id}\,}}_1$ is the least element under computable reducibility (in ceers, co-ceers, and ${\mathcal {X}}$).
2. ${{\,\mathrm{Id}\,}}_2$ is the unique minimal *c*-degree over ${{\,\mathrm{Id}\,}}_1$ (in ceers, co-ceers, and ${\mathcal {X}}$).
3. Inside ${\mathcal {X}}$, one can find two incomparable elements $x_0$ and $x_1$ (namely, the *c*-degrees of ${{\,\mathrm{Id}\,}}_3$ and $F_W$) such that ${{\,\mathrm{Id}\,}}_2 < x_i$ and $\lnot \exists z ( {{\,\mathrm{Id}\,}}_2< z < x_i )$. Note that this property fails for ceers and co-ceers.

The degree structure of ${\mathcal {X}}$-equivalence relations is elementary equivalent to the degree structure of neither ceers nor co-ceers. $\square$

### Finite minimality

We move now to equivalence relations with infinitely many equivalence classes.

Notice that in the context of ceers, explored in [3], equivalence relations with finitely many classes are computable, whereas this is not so in higher levels of the Ershov hierarchy as witnessed by the two-classes equivalence relations of the form $F_X$. This suggests the following notion of minimality (called finite minimality) when we work in the Ershov hierarchy.

#### Definition 2.2

An equivalence relation *R* is *finitely minimal* if $S<_c R$ implies that *S* has only finitely many equivalence classes.

Observe also that if *R* is light and $R \not \leqslant _c {{\,\mathrm{Id}\,}}$, then *R* is not finitely minimal.

The previous definition does perfect justice to the notion of minimality for dark equivalence relations, as it is easy to see (see [3] where the property is stated for ceers, but it clearly holds of all equivalence relations) that if *E* is dark, $R \leqslant _c E$ and *R* has infinitely many equivalence classes, then *R* is dark as well. So a finitely minimal dark equivalence relation is exactly a minimal dark equivalence relation, i.e. a dark equivalence relation for which there is no dark equivalence relation strictly below it.

One of the tools that will be useful to us is the *collapse technique*, extensively used for ceers in [3]. If *R* is an equivalence relation and , then the *collapse* $R_{{{\,\mathrm{coll}\,}}(x,y)}$ (denoted by $R_{/(x,y)}$ in [3]) is defined as

$$\begin{aligned} R_{{{\,\mathrm{coll}\,}}(x,y)} := R \cup \{ (u,v) \,:u,v \in [x]_R \cup [y]_R\}. \end{aligned}$$

We illustrate the technique by obtaining the next two results about finite minimality.

#### Lemma 2.3

Suppose that *R* is dark with a computable class. Then *R* cannot be finitely minimal.

#### Proof

Suppose that classes $[a]_R$ and $[b]_R$ are distinct, and $[b]_R$ is computable. Consider the collapse $S := R_{{{\,\mathrm{coll}\,}}(a,b)}$. Then (by essentially the same argument as in the proof of [3, Lemma 2.6]) *S* is reducible to *R* by the function

$$\begin{aligned} f(x) = \left\{ \begin{array}{ll} x, &{}\quad \text {if } x\not \in [b]_R,\\ a, &{}\quad \text {otherwise}. \end{array} \right. \end{aligned}$$

Assume that $g:R\leqslant _c S$. Consider the map $h := f\circ g$ and the *h-orbit* of *b*, i.e. the set

$$\begin{aligned} {{\,\mathrm{orb}\,}}_h(b) = \{ h^k(b) \,:k\in \omega \}. \end{aligned}$$

It is easy to see that $h:R\leqslant _c R$. We claim that the orbit ${{\,\mathrm{orb}\,}}_h(b)$ consists of pairwise non-*R*-equivalent elements: indeed, if  for $k<l$, then we have , and ${{\,\mathrm{range}\,}}(f) \cap [b]_R\ne \emptyset$, which contradicts the choice of the map *f*. Hence, ${{\,\mathrm{orb}\,}}_h(b)$ is a c.e. transversal of *R*, and we obtain a contradiction with the darkness of *R*. Therefore, we deduce that $S<_c R$ and *R* is not finitely minimal. $\square$

Now we show that there are infinitely many levels of the Ershov hierarchy which contain finitely minimal dark equivalence relations *properly* belonging to the level.

#### Theorem 2.4

Suppose that $a\in {\mathcal {O}}$ and $|a|_{{\mathcal {O}}} \geqslant 1$. Then there exists a finitely minimal, dark equivalence relation $R \in \Delta ^0_2 \smallsetminus \Sigma ^{-1}_a$.

#### Proof

In the proof of [3, Theorem 3.3], Andrews and Sorbi constructed infinitely many pairwise incomparable finitely minimal dark ceers $S_l$, $l\in \omega$, with the following property: for any *e* and *l*, if the c.e. set $W_e$ intersects infinitely many $S_l$-classes, then it intersects *every* $S_l$-class. We choose only one such ceer $S := S_0$. As explained in the introduction, fix a $\Sigma ^{-1}_a$ list $(E_e)_{e\in \omega }$ of all $\Sigma ^{-1}_a$ sets $(E_e)_{e\in \omega }$. We build a $\Delta ^0_2$ equivalence relation *R* with the following properties:

- $$R\supseteq S$$, and
- $$R\ne E_e$$, for every $$e\in \omega $$.

The construction proceeds in a straightforward ${\mathbf {0}}'$-effective manner. We choose a ${\mathbf {0}}'$-effective list $\{ x_e\}_{e\in \omega }$ which enumerates representatives of all *S*-classes, without repetitions (i.e.  for $i\ne j$). We start with $R[0]=S$, i.e. *S* is the approximation *R*[0] to *R* at stage 0.

Consider stage *k*. If , then $R[k+1]$ is equal to the collapse $R[k]_{{{\,\mathrm{coll}\,}}(x_{2k},x_{2k+1})}$. Otherwise, $R[k+1] = R[k]$.

As per usual, set $R = \bigcup _{k\in \omega } R[k]$. First notice that *R* is $\Delta ^0_2$: to see if  use oracle ${\mathbf {0}}'$ to search for the unique *h*, *k* such that  and , and then check if $x_h=x_k$ or $x_h$ and $x_k$ have been collapsed at stage $[\frac{h}{2}]$. Finally, it is not hard to show that *R* has infinitely many classes, and $R\not \in \Sigma ^{-1}_a$.

Assume now that $f:Id\leqslant _c R$. Since $R\supseteq S$, the map *f* is also a reduction from *Id* to *S*, which contradicts the darkness of *S*. Thus, *R* is also dark.

Suppose that $g:Q\leqslant _c R$ and *Q* has infinitely many classes. Since *Q* contains infinitely many classes, the set $W := {{\,\mathrm{range}\,}}(g)$ is a c.e. set which intersects infinitely many *R*-classes. Recall that $R\supseteq S$, hence, *W* intersects infinitely many *S*-classes. The choice of the ceer *S* implies that *W* intersects every *S*-class.

Now we build a map *h* as follows: fix an effective approximation $\{ S[t]\}_{t\in \omega }$ of the ceer *S* and for a number *x*, define *h*(*x*) to be the first seen *y* such that : more formally,

$$\begin{aligned} t(x) := \mu t [ (\exists y\leqslant t) ( (x, g(y)) \in S[t] ) ],\\ h(x) := \mu y [(x, g(y)) \in S[t(x)]]. \end{aligned}$$

Since ${{\,\mathrm{range}\,}}(g)$ intersects every *S*-class, *h* is a computable function. We show that $h:R\leqslant _c Q$. Note that for any $x,y\in \omega$, we have  and the following conditions are equivalent:Thus, we have $Q\equiv _c R$, and *R* is finitely minimal. Theorem 2.4 is proved. $\square$

Note that, in the relation *R* from the theorem above, every *R*-class is a c.e. set. Thus, *R* has the following curious property: if $Q\leqslant _c R$ and *Q* has only finitely many classes, then $Q\equiv _c Id_n$ for some $n\in \omega$.

### Failure of inversion lemma

A fundamental technique when studying ceers is provided by the following result: see for instance [3, Lemma 1.1].

#### Lemma 2.5

(Inversion lemma). Suppose that *R*, *S* are ceers and $R\leqslant _c S$ via *f*. If *f* hits all the equivalence classes of $S\ ($i.e. ${{\,\mathrm{range}\,}}(f)$ intersects *every* *S*-class), then $S\leqslant _c R$.

The Inversion lemma does not hold, in general, for the Ershov hierarchy. In fact, it fails already for 2-ceers:

#### Lemma 2.6

There is an equivalence relation $R\in \Sigma ^{-1}_{2} \smallsetminus \Sigma ^{-1}_{1}$ such that $Id\leqslant _c R$ via a function *f* which is surjective on the equivalence classes of *R*.

#### Proof

Fix a noncomputable c.e. set *X*. Split $\omega$ into four computable parts: $A = \{ a_0,a_1,a_2,\dots \}$, $B \!=\! \{ b_0, b_1, b_2, \dots \}$, $C \!= \!\{ c_0, c_1, c_2,\dots \}$, and $D \!=\! \{ d_0,d_1,d_2,\dots \}$.

The relation *R* is given by its equivalence classes: for every $i\in \omega$,

- If $i\not \in X$, then *R* contains disjoint classes $\{ a_i, c_i\}$ and $\{ b_i, d_i\}$.
- If $i\in X$, then there are *R*-classes $\{ a_i, d_i\}$ and $\{ b_i, c_i\}$.

It is clear that *R* is not a ceer, and the function

$$\begin{aligned} f(2i) = a_i, \quad f(2i+1) = b_i, \end{aligned}$$

gives a reduction from *Id* to *R*, hitting all the *R*-classes. Furthermore, *R* is a 2-ceer, since after separating two classes in the approximation of *R* (due to some *i* being enumerated in *X*), we never merge them again. $\square$

#### Lemma 2.7

For any dark $\Delta ^0_2$ equivalence relation *R*, there are a dark equivalence relation $R<_c S$ and a reduction $f: R\leqslant _c S$ such that *f* hits all the *S*-classes.

#### Proof

Fix a dark ceer *Q*. Choose a ${\mathbf {0}}'$-effective list $\{ r_i \}_{i\in \omega }$ of representatives of all *R*-classes, without repetitions. Similarly, choose a ${\mathbf {0}}'$-effective list $\{ q_i\}_{i\in \omega }$ for representatives of *Q*-classes.

The construction is given in a ${\mathbf {0}}'$-computable way. At stage 0, set $S[0] := R\oplus Q$. At stage $e+1$, if , then set $S[e+1] := S[e]_{{{\,\mathrm{coll}\,}}(2r_e,2q_e+1)}$. Otherwise, define $S[e+1]:=S[e]_{{{\,\mathrm{coll}\,}}(0,2q_e+1)}$.

It is not hard to show that the constructed *S* is $\Delta ^0_2$, and $S\nleqslant _c R$. Moreover, the function $f:x\mapsto 2x$ gives a reduction of *R* to *S*, hitting all the *S*-classes.

Assume that $g:Id\leqslant _c S$. Then either ${{\,\mathrm{range}\,}}(g)$ contains infinitely many even numbers or infinitely many odd numbers. In any case, this leads to a contradiction with darkness of *R* or *Q*, thus, *S* is dark. $\square$

## Dark equivalence relations in the Ershov hierarchy

In this section, we show that the phenomenon of darkness is rather pervasive: with the exception of the co-ceers, dark degrees exist properly at each level of the Ershov hierarchy. In the construction of these degrees we also develop some strategies that will be helpful in further sections, when the focus will be in on the existence of infima and suprema of *c*-degrees.

### Proposition 3.1

There are no dark co-ceers.

### Proof

Suppose that $R\in \Pi ^0_1$. If *R* has finitely many equivalence classes, then it is trivially not dark. Hence, assume there exist infinitely many *R*-classes. We prove that *R* is light by inductively building the following c.e. transversal of *R*: Let $x_0=0$ and let $x_{i+1}$ be any number *z* such that, for all $j\leqslant i$, $(x_j,z)\notin R$. Such a *z* must exist (otherwise, there would be only finitely many *R*-classes) and, since *R* is co-c.e.  will be found effectively. $\square$

### Theorem 3.2

If ${\mathcal {X}} \in \{\Sigma ^{-1}_a: a \in {\mathcal {O}}, |a|_{\mathcal {O}}\geqslant 1\} \cup \{\Pi ^{-1}_a: a \in {\mathcal {O}}, |a|_{\mathcal {O}}>1\}$ then there is a dark equivalence relation having only finite equivalence classes, and properly lying in ${\mathcal {X}}$.

### Remark 3.3

Theorem 3.2 can be obtained as a corollary of Theorem 4.6. For the sake of exposition, being the proof of Theorem 4.6 more complicated, we shall provide a proof of Theorem 3.2 nonetheless. This might help the reader to familiarize, in a simpler context, with the techniques required for Theorem 4.6.

### Proof of Theorem 3.2

Let us start with the case ${\mathcal {X}}=\Sigma ^{-1}_a$, $|a|_{\mathcal {O}}\geqslant 1$. We want to build an equivalence relation *R* satisfying the following requirements, for every $e \in \omega$:

$$\begin{aligned}&F_{e}: [e]_{R} \text { is finite,}\\&P_e: W_e \text { is not a transversal for } R, \\&Q_e: R \ne E_e, \end{aligned}$$

where $(E_e)_{e \in \omega }$ is a listing of the $\Pi ^{-1}_a$ sets as explained in the introduction, with $\langle f_{e}, \gamma _{e}\rangle$ a $\Pi ^{-1}_a$-approximation pair to $E_{e}$ satisfying also (*).

Let us define the *priority ordering* of the requirements as

$$\begin{aligned} F_{0}<Q_0<P_0< \cdots< F_{e}< Q_e< P_e < \cdots . \end{aligned}$$

We build computable functions *f*(*x*, *s*) and $\gamma (x,s)$ so that the pair $\langle f, \gamma \rangle$ is a $\Sigma ^{-1}_a$-approximation pair (in the sense of Definition 1.4) to a $\Sigma ^{-1}_a$ equivalence relation *R* defined as

$$\begin{aligned} x \mathrel {R} y \Leftrightarrow \lim _s f(\langle x, y \rangle ,s)=1, \end{aligned}$$

satisfying the given requirements. In fact at step *s* we define a computable set *R*[*s*], where $x \mathrel {R[s]} y \Leftrightarrow f(\langle x, y \rangle ,s)=1$, and thus $x \mathrel {R} y$ if and only if  for cofinitely many *s*. Except for *R*[0], which is the empty set, for every $s>0$ *R*[*s*] is an equivalence relation in which almost all equivalence classes are singletons. Notice that we do not define here $R[0]={{\,\mathrm{Id}\,}}$, as would be perhaps more appropriate by the reflexivity property of equivalence relations, because we want to accompany the definition of *R* with an accompanying $\Sigma ^{-1}_a$-approximation pair $\langle f, \gamma \rangle$ to *R* so that *f* must start with $f(x,0)=0$, for every *x*.

*Strategies for the requirements and their interactions* The strategy for $F_{e}$ requires that no lower priority requirement modify $[e]_{R}$, which is therefore eventually finite because only higher priority requirements may contribute with their actions to add elements to $[e]_{R}$ (we will see that every requirement may act only finitely many times).

For the *Q*-requirement $Q_{e}$ we appoint as witness a pair $(x_e, y_e)$, with $(x_{e}, y_{e}) \in 2\omega \times (2\omega +1)$: we will in fact choose $(x_e, y_e)$ of the form $(x_e, y_e)=(2i, 2i+1)$ for some *i*. The reason we choose $x_e$ to be even and $y_e$ to be odd is for the sake of *P*-requirements. The requirement $P_e$ waits for $W_{e}$ to enumerate a pair of distinct numbers *u*, *v*, both even, or both odd, which avoid the finitely many classes restrained by higher priority requirements. When found, it simply *R*-collapses *u*, *v* (if not already collapsed): this ensures that $W_{e}$ is not a transversal for *R*. Notice that if $W_e$ is infinite then by the Pigeon Hole Principle it either contains infinitely many even numbers or infinitely many odd numbers. We will see by Lemma 3.4 that $P_e$ will not be restrained by $\Sigma ^{-1}_{a}$-ness from *R*-collapsing two even numbers or two odd numbers, since the construction will ensure that a necessary condition, at any stage *s*, for which we may have $f(\langle x,y\rangle , s)=0$ but already $\gamma (\langle x,y\rangle , s)=1$, is that *x*, *y* have different parity.

When at a stage $s_0$ we appoint $(x_e,y_e)$, we may assume by (*) that $f_{e}(\langle x_{e}, y_{e} \rangle ,s_0)=1$ and $\gamma _{e}(\langle x_{e}, y_{e} \rangle ,s_0)=a$: so we start up with having $(x_{e}, y_{e})$ not in *R*, i.e., $f(\langle x_{e}, y_{e} \rangle ,s_0)=0$, and $\gamma (\langle x_{e}, y_{e} \rangle ,s_0)=a$. Every time we see, after this, $f_{e}(\langle x_{e}, y_{e} \rangle ,s+1)\ne f_e(\langle x_{e}, y_{e} \rangle ,s)$ , we change accordingly $f(\langle x_{e}, y_{e} \rangle ,s+1)$ as to have

$$\begin{aligned} f(\langle x_{e}, y_{e} \rangle , s+1) \ne f_{e}(\langle x_{e}, y_{e} \rangle ,s+1), \end{aligned}$$

and we define

$$\begin{aligned} \gamma (\langle x_{e}, y_{e} \rangle ,s+1):= \gamma _{e}(\langle x_{e}, y_{e} \rangle , s+1). \end{aligned}$$

In this way $Q_{e}$ is able to diagonalize against $E_{e}$ at the witness $(x_{e}, y_{e})$, consistently with *R* being in $\Sigma ^{-1}_{a}$. When we choose $x_{e}$ and $y_{e}$ we choose them big enough so that the corresponding finiteness requirements $F_{x_{e}}$ and $F_{y_{e}}$ follow $Q_{e}$ in the priority listing of the requirements, so that they do not impose any restraint to $Q_{e}$, which of course sets up a restraint requiring that no lower priority requirement modify the equivalence classes of $x_e$ and $y_e$.

*The construction* The construction is in stages: at stage *s* we define the approximation *R*[*s*] to *R*, and the approximations to the various parameters $(x_{e}, y_{e})$. We will often omit to mention the stage to which a given parameter is referred, if this is clear from the context. Unless otherwise specified, at each stage each parameter keeps the same value as at the previous stage. To *initialize* a *Q*-requirement at stage *s* means to set as undefined at that stage the current value of its witness. A pair $(x_e,y_e)$ is an *active* witness for $Q_e$ at stage *s* if the pair has been appointed as a witness for $Q_e$ at some previous stage, and has never been canceled thereafter. We say that a requirement $P_e$ is *inactive* at the end of stage *s* if there are already distinct numbers $u,v \in W_e$ such that  at the end of stage *s*; it is *active* otherwise.

A requirement *T* *requires attention at stage* $s+1$ if either

1. *T* is initialized; or
2. one of the following holds, for some *e*:
  - $T=P_e$, $P_e$ is active at the end of *s*, and at the current stage there is a pair of distinct numbers $u,v \in W_{e}$ both even or both odd, and *u*, *v* bigger than all numbers in the union of all current equivalence classes of numbers $i \leqslant e$, and $x_i, y_i$, for $i\leqslant e$ (in this way, *u*, *v* are respectful of the restraint imposed by higher priority requirements);
  - $T=Q_e$ and $f(\langle x_e, y_e\rangle ,s)= f_e(\langle x_e, y_e\rangle ,s+1)$, where $(x_e,y_e)$ is the witness of *T* at the end of stage *s*.

*Stage* 0 Initialize all *Q*-requirements. Define $f(x,0):=0$ and $\gamma (x,0):=a$ for all *x*; consequently $R[0]= \emptyset$.

*Stage* 1 Define $f(\langle x, x \rangle , 1):=1$ and $\gamma (\langle x, x \rangle , 1):=1$ for all *x*, leaving $f(\_,1):=f(\_,0)$ and $\gamma (\_,1):=\gamma (\_,0)$ the other values of *f* and $\gamma$. Consequently, $R[1]= {{\,\mathrm{Id}\,}}$. [Recall that $|1|_{{\mathcal {O}}}=0$. Notice that for every $s \geqslant 1$, we will have , so there will never be need to redefine $\gamma (\langle x, x \rangle , \_)$.] Therefore we can say that the construction essentially starts (with $R={{\,\mathrm{Id}\,}}$) at stage 1 instead of 0.

*Stage* $s+1\geqslant 2$ Consider the highest priority requirement *T* that requires attention. (Notice that there is always such a requirement since at each stage almost all *Q*-requirements are initialized.) Action:

1. If *T* is initialized, then $T=Q_e$ for some *e*: choose a fresh witness $(x_e, y_e)$ for *T*, i.e. $x_e=2i$ and $y_e=2i+1$, where *i* is bigger than all numbers so far used in the construction (either as components of witnesses, or as numbers involved in collapses demanded by *P*-requirements, bigger than all numbers in the current equivalence classes $[j]_R$, for $j \leqslant e$, and so that $Q_{e}$ precedes $F_{2i}$ and $F_{2i+1}$ in the priority listing of the requirements). Notice that because of (*) in the definition of a $\Pi ^{-1}_a$-approximation, we may as well suppose that still $f_e(\langle x_e, y_e \rangle ,s+1)=1$, $f(\langle x_e, y_e \rangle ,s)=0$, and $\gamma _e(\langle x_e, y_e \rangle ,s+1)=\gamma (\langle x_e, y_e \rangle ,s)=a$.
2. Otherwise:
  - if $T=P_e$ then pick the least pair *u*, *v* as in the definition of requiring attention; define, for any $x,y\in [u]_{R[s]} \cup [v]_{R[s]}$, $f(\langle x, y\rangle , s+1):=1$ and $\gamma (\langle x, y\rangle , s+1):=1$;
  - if $T=Q_e$ then define $f(\langle x_e, y_e \rangle ,s+1)$ so that $f(\langle x_e, y_e \rangle ,s+1)\ne f_e(\langle x_e, y_e \rangle ,s+1)$, and $\gamma (\langle x_e, y_e\rangle , s+1):=\gamma _e(\langle x_e, y_e\rangle , s+1)$, so that we diagonalize *R* against $E_e$. [Notice that by Lemma 3.4, $f(\_,s+1)$ will still the characteristic function of an equivalence relation.]

$R[s+1]$ is consequently the equivalence relation having $f(\_,s+1)$ as characteristic function. This is the end of the stage. Initialize all lower priority *Q*-requirements.

*The verification* An easy inductive argument shows that for every requirement *T* there is a least stage $t_{T}$ such that no $T'$ of higher priority than *T*, nor *T* itself, requires attention or acts at any $s \geqslant t_{T}$. Indeed suppose that this is true for every $T'$ of higher priority than *T*. Then there is a least stage *t* such that no such $T'$ requires attention after *t*. So either $T=P_e$ and thus *T* may act at most once after *t*; or $T=Q_e$ and thus *T* may require attention a first time to appoint the final value of its witness $(x_e,y_e)$ (this value will never be canceled, because *T* will never be re-initialized again, as no higher priority $T'$ will ever act again), and subsequently finitely many times in response to the finitely many changes of $f_e(\langle x_e,y_e\rangle ,s)$. In any case this shows that $t_{T}$ exists.

### Lemma 3.4

For every *e*, *s*, if $(x_e,y_e)$ is still an active witness for $Q_e$ at *s*, then at that stage $[x_e]_R \cup [y_e]_R = \{x_e,y_e\}$, each equivalence class among $[x_e]_R$ and $[y_e]_R$ being a singleton if and only if $f_{e}(\langle x_e,y_e \rangle ,s)=1$. Moreover, if distinct *u*, *v* have the same parity then $f(\langle u,v\rangle , \_)$ may change at most once from value 0 to 1 in response to the action of some *P*-requirement which becomes inactive.

### Proof

Suppose that $(x_e,y_e)$ is active at *s*, and let $t\leqslant s$ be the stage at which this witness has been appointed for $Q_e$. Then the restraint imposed by $Q_e$ prohibits any modification of the equivalence classes of $x_e, y_e$ done by any lower priority requirement. Such a modification can only be performed by a higher priority requirement $P_i$, but if such a requirement has acted after *t*, then the witness $(x_e,y_e)$ has been re-initialized and thus canceled.

The latter claim about the number of changes of $f(\langle u,v\rangle ,\_)$ if *u*, *v* are distinct and have the same parity follows from the fact that by the first part of this lemma the value $f(\langle u,v\rangle ,\_)$ is not changed by any *Q*-requirement, so if the value $f(\langle u,v\rangle ,1)=0$ is later changed from 0 to 1 then this is due to a *P*-requirement, which becomes inactive, and by choice of *u*, *v* and the first part of this lemma this change is never revoked by any higher priority *Q*-requirement. $\square$

This enables us to show also that *T* is in the end satisfied. This claim is evident if $T=F_{e}$ as after $t_{Q_{e}}$ no *P*-requirement can add numbers to $[e]_{R}$ because of the restraint imposed by $F_e$. This shows that each *R*-equivalence class is finite.

The claim is also evident if $T=Q_e$ as *T* is by Lemma 3.4 the only requirement which is entitled to move its final witness $(x_e,y_e)$ in or out of *R*.

To show that *T* is satisfied if $T=P_e$, assume that $W_e$ is infinite. Since the witness $(x_{e}, y_{e})$ reaches a limit, and all equivalence classes are finite, it follows that $W_{e}$ contains at least two even numbers respectful (as specified in the definition of $P_e$ requiring attention) of the restraint imposed by higher priority requirements, so that at some point we are able to *R*-collapse such a pair *u*, *v* if $P_{e}$ is still active. So if $W_e$ is infinite then it can not be a transversal of *R*.

By Lemma 3.4 it is also clear that $R\in \Sigma ^{-1}_{a}$, and the pair $\langle f, \gamma \rangle$ is a $\Sigma ^{-1}_{a}$-approximation to *R*. Indeed, any action at stage $s+1$ which makes $f(x,s+1)\ne f(x,s)$ is accompanied by a corresponding definition of $\gamma$ which makes $\gamma (x,s+1) <_{{\mathcal {O}}} \gamma (x,s)$. For instance, if we act due to (2a) then $\gamma (\langle u, v\rangle , s)=a$, hence $\gamma (\langle u, v\rangle , s+1) =1 <_{{\mathcal {O}}} a= \gamma (\langle u, v\rangle , s)$.

Finally, we consider the case when ${\mathcal {X}}=\Pi ^{-1}_a$ for some notation *a* with $|a|_{{\mathcal {O}}}>1$. The construction is virtually the same as in the dual case. We start with $R[0]={{\,\mathrm{Id}\,}}_1$ by defining $f(x, 0):=1$ and $\gamma (x, 0):=a$ for every *x*. Next, we take *R*[1],[recall that we pick witnesses $(x_e, y_e)$ to be of the form $(2i, 2i+1)$] by suitably defining *f* and $\gamma$: in particular

$$\begin{aligned} \gamma (\langle x, y\rangle ,1)= {\left\{ \begin{array}{ll} a, &{}\text {if } x=y \vee (\exists i)[\{x,y\}=\{2i, 2i+1\}],\\ 2, &{}\text {otherwise,} \end{array}\right. } \end{aligned}$$

where we have used the assumption that $|a|_{{\mathcal {O}}}>1$. The construction now mimics the one for the $\Sigma ^{-1}_a$ case, essentially starting from stage 1. The idea is to make the first non-trivial approximation *R*[1] to *R* to look very much like ${{\,\mathrm{Id}\,}}$ (as in the proof of Theorem 3.2), except for pairs of the form $(2i, 2i+1)$ for which we do not want to add an extra initial change which could spoil our possibility of playing the extraction/enumeration game exploited by the diagonalization strategies. This allows a requirement $P_e$ to be able, if it requires attention, to *R*-collapse pairs of numbers of the same parity that have been set as non-*R*-equivalent at stage 1 (when we have defined $\gamma$ to be 2 for these pairs). $\square$

## The problem of the existence of infima

Let us fix a notation *a* for a non-zero computable ordinal. The following observation is straightforward.

### Fact 4.1

The poset of degrees of $\Sigma ^{-1}_a$ equivalence relations is not a lower-semilattice.

### Proof

This follows immediately from the fact that the degrees of ceers forms an initial segment of the degrees of $\Sigma ^{-1}_a$ equivalence relations, and on the other hand ceers are known not to form a lower-semilattice, see for instance [3]. $\square$

We will show however that we can find properly $\Sigma ^{-1}_a$ equivalence relations with no $\inf$, proving that the *c*-degrees of $\Sigma ^{-1}_a\smallsetminus \Pi ^{-1}_a$ equivalence relations do not form a lower-semilattice.

Andrews and Sorbi [3] showed that many questions about the degree structure of ceers can be fruitfully tackled by inspecting the interplay between light and dark ceers. By lifting our focus to the class of $\Delta ^0_2$ equivalence relations, we need to introduce the following relativized version of Definition 1.3, which stands as a natural companion of the analysis of the complexity of transversals of a given ceer provided in [14].

### Definition 4.2

Let *R* be an equivalence relation, and *A* any set of numbers. *R* is *A-dark* if *R* is infinite and has no *A-transversal*, i.e. there is no infinite *A*-c.e. set $W_e^A$ such that, for all distinct $u,v \in W_e^A$, one has .

Two equivalence relations *R*, *S* are *mutually dark* if *R* is *S*-dark, and *S* is *R*-dark.

Notice.

### Proposition 4.3

If *R*, *S* are mutually dark then they are dark.

### Proof

In fact, if *R* is such that there is a set *A* such that *R* has no *A*-c.e. set as a transversal then *R* is dark. This follows from the fact that a c.e. set is *A*-c.e. relatively to every oracle *A*. $\square$

By Theorem 4.6, we prove that mutually dark equivalence relations exist at all levels of the Ershov hierarchy. But before that, let us offer a nice alternative characterization of mutual darkness. We first need to relativize computable reducibility in an obvious way (as in [14], where the complexity of ${\mathbf {d}}$-computable reductions is considerably explored): Let ${\mathbf {d}}$ be a Turing degree. *R* is ${\mathbf {d}}$*-computably reducible* to *S*, denoted $R\leqslant _{{\mathbf {d}}} S$, if there is a total ${\mathbf {d}}$-computable function *f* such that, for all $x,y\in \omega$,

### Definition 4.4

Define $R|_d S$ if *R*, *S* are infinite and $R\nleqslant _{\deg _T(R)} S$ and $S\nleqslant _{\deg _T(S)} R$.

Hence, $R|_d S$ holds if the information of neither of the two equivalence relations is enough by itself to compute a reduction into the other. The next proposition shows that this is the same as asking that *R* and *S* are mutually dark.

### Proposition 4.5

$R |_d S$ if and only if *R*, *S* are mutually dark.

### Proof

Let *R*, *S* be infinite equivalence relations.

Suppose that *R* is not *S*-dark, and let *g* be an *S*-computable function which lists a transversal of *R*. We claim in this case that $S \leqslant _{\deg _{T}(S)} R$. Indeed, a suitable *S*-computable function reducing *S* to *R* can be defined by induction as follows: $f(0):=g(0)$; andIn a similar way one can show that if *S* is not *R*-dark then $R \leqslant _{\deg _{T}(R)} S$.

Vice versa suppose that $S \leqslant _{\deg _{T}(S)} R$, via a function $f \in \deg _{T}(S)$. As *S* is infinite, let *g* be an *S*-computable function listing a transversal for *S*. Then the function $f \circ g$ is *S*-computable and lists a transversal of *R*. Thus *R* is not *S*-dark. In a similar way, one shows that if $R \leqslant _{\deg _{T}(R)} S$ then *S* is not *R*-dark. $\square$

### Theorem 4.6

If ${\mathcal {X}} \in \{\Sigma ^{-1}_a: a \in {\mathcal {O}}, |a|_{\mathcal {O}}\geqslant 1\}\cup \{\Pi ^{-1}_a: a \in {\mathcal {O}}, |a|_{\mathcal {O}}>1\}$ then there exist mutually dark equivalence relations having only finite classes, and properly lying in ${\mathcal {X}}$.

### Proof

Let us start again with the case ${\mathcal {X}}=\Sigma ^{-1}_a$, with $|a|_{\mathcal {O}}\geqslant 1$. We build equivalence relations *U*, *V* satisfying the following requirements, for every $e \in \omega$:

$$\begin{aligned}&F^{U}_{e}: [e]_{U} \text { is finite,}\\&F^{V}_{e}: [e]_{V} \text { is finite,}\\&P^U_e: W_e^U \text { is not a transversal for } V,\\&P^V_e: W_e^V \text { is not a transversal for } U,\\&Q^U_e: U \ne E_e,\\&Q^V_e: V \ne E_e, \end{aligned}$$

where $(E_e)_{e \in \omega }$ is an effective listing of the $\Pi ^{-1}_a$ sets, with $\langle f_{e}, \gamma _{e}\rangle$ a $\Pi ^{-1}_a$-approximation pair to $E_{e}$, satisfying (*). We build *U*, *V* via defining $\Sigma ^{-1}_a$-approximation pairs $\langle f^U, \gamma ^U \rangle$ and $\langle f^V, \gamma ^V \rangle$ to *U*, *V* respectively.

The *priority ordering* of the requirements is

$$\begin{aligned}&F_{0}^{U}< F_{0}^{V}<Q_0^U< Q_0^V<P_0^U< P_0^V< \cdots \\&\quad< F_{e}^{U}< F_{e}^{V}< Q_e^U< Q^V_e< P^{U}_e< P_e^V < \cdots . \end{aligned}$$

*Strategies for the requirements and their interactions* The strategies for the requirements are essentially the same as the ones for the “corresponding” requirements in the proof of Theorem 3.2. The additional complication is due to the fact that we have also sometimes to preserve certain Turing-computations. For this reason we will view the restraint imposed at any stage by a *P*-requirement *R* as a finite binary string $r_{R,U}$ or $r_{R,V}$: if $S \in \{U,V\}$ the string $r_{R,S}$ extends $r^-_{R,S}$ (i.e. $r_{R,S} \supseteq r^-_{R,S}$) which represents the restraint coming at that stage from the higher priority *P*-requirements (the string $r^-_{R,S}$ is empty if *R* is not preceded in the priority ordering by any higher priority *P*-requirement); in turn, $r_{R,S}$ is a string which lower priority requirements are bound to preserve if *R* is not re-initialized: this string automatically becomes $r_{R,S} = r^-_{R',S}$ where $R'$ is the requirement immediately following *R* in the priority ordering. The strings $r^-_{R,S}$ and $r_{R,S}$ depend of course on the stage: we denote by $r^-_{R,S}(s)$ and $r_{R,S}(s)$ their values at stage *s*.

The strategy for $R=F^U_e$ (the one for $F^V_e$ is similar) sets up a restraint requesting that no lower priority requirement change $[e]_{U}$.

The strategy for $R=Q^U_e$ (the one for $Q^V_e$ is similar) works with a suitable witness $(x^U_e, y^U_e) \in 2\omega \times (2 \omega +1)$, consisting as in the proof of Theorem 3.2 of numbers of the form $(2i,2i+1)$, and sets up a restraint to preserve the *U*-equivalence classes of $x^U_e$ and $y^U_e$. We assume we have chosen 2*i* and $2i+1$, so that $Q^U_e$ precedes $F_{2i}^{U}$ and $F_{2i+1}^{U}$ in the priority listing of the requirements, so that the latter two requirements do not impose any restraint to $Q_{e}^{U}$.

Let us now consider a *P*-requirement $R=P_e^U$ (the case $R=P_{e}^{V}$ is similar). The strategy for $P^U_e$ consists in seeing if we can define *U*, *V* so that there are distinct $u,v\in W_e^{U}$ of the same parity, and *u*, *v* can be *V*-collapsed without injuring higher priority restraints. It sets up a restraint asking that the initial segment $r_{R,U}$ of the characteristic function of *U*, sufficient to keep $u,v\in W_e^{U}$, be preserved.

Due to the action of higher priority requirements, *P*- and *Q*-requirements may have to be (re-)*initialized* as in the proof of Theorem 3.2, by setting their parameters as undefined (in the case of a $P^{S}$-requirement this entails that all $r_{R',S}$ are set to be undefined, for all lower priority $R'$); moreover, as in that proof, when at any stage $s+1$ a $P^U$-requirement *V*-collapses a pair of distinct numbers, then this collapse is forever, reflected in the fact that we define $\gamma ^V(\langle u, v \rangle ,s+1)=1$: the same applies of course for the symmetric case, where the roles of *U* and *V* are interchanged.

A requirement *R* *requires attention at stage* $s+1$ if either

1. *R* is initialized and *R* is a *Q*-requirement; or
2. one of the following holds, with $e \in \omega$ and $S \in \{U,V\}$ (we assume for definiteness $S=U$, the other case being similar and treated by interchanging the roles of *U* and *V*):
  - $R=P_e^U$, *R* is *active* at the end of *s* (i.e. it is not the case that at $s+1$, $r_{R,U}$ has been already defined and there are already distinct numbers *u*, *v* with $u,v \in W_e^{r_{R,U}}$ and ), and there are now a string $\sigma$ and a pair (*u*, *v*) of distinct numbers of the same parity such that:
    (i) $r^{-}_{R,U} \subseteq \sigma \subset U[s]$ (the latter inclusion means that $\sigma$ is an initial segment of the characteristic function of *U*[*s*]), and $u,v\in W_e^{\sigma }$;
    (ii) $V[s]_{{{\,\mathrm{coll}\,}}{(u,v)}}$ and *V*[*s*] give the same equivalence classes relatively to the *V*-equivalence classes restrained by higher priority $F^V$- and $Q^V$-requirements, and the *V*-collapse of the equivalence classes of *u*, *v* does not alter $r^-_{R,V}$.
  - $R=Q_e^U$ and $f^U(\langle x_e^U, y_e^U\rangle ,s)= f_e(\langle x_e^U, y_e^U\rangle ,s+1)$, where $(x_e^U,y_e^U)$ is the witness of *R* at the end of stage *s*.

*The construction* At stage *s* we define approximations to $f^{S}(\_,s)$ and $\gamma ^{S}(\_,s)$ for $S \in \{U,V\}$ (if $s>0$ then *U*[*s*] and *V*[*s*] will be the equivalence relations having $f^{U}(\_,s)$ and $f^{V}(\_,s)$ as characteristic functions, respectively: in this case, it will be clear from the construction that *U*[*s*] and *V*[*s*] are equivalence relations with only finite equivalence classes and such that almost all equivalence classes are singletons; on the other hand we define $S[0]:=\emptyset$), and the approximations to the various parameters, including $r_{R,S}$ and $r^{-}_{R,S}$. We will often omit to mention the stage to which a given parameter is referred, if this is clear from the context. Unless otherwise specified at each stage each parameter keeps the same values as at the previous stage.

*Stage* 0 Initialize all *P*- and *Q*-requirements; for $S\in \{U,V\}$ define $f^{S}(x,0):=0$ and $\gamma ^{S}(x,0):=a$ for all *x*; consequently $S[0] = \emptyset$.

*Stage* 1 For $S\in \{U,V\}$ define $f^S(\langle x, x \rangle , 1):=1$ and $\gamma ^S(\langle x, x \rangle , 1):=1$ for all *x*, keeping the values already defined at 0 for the other values of both $f^S(\_,1)$ and $\gamma ^S(\_,1)$. Consequently, $S[1]= {{\,\mathrm{Id}\,}}$.

*Stage* $s+1\geqslant 2$ Consider the highest priority requirement *R* that requires attention. (Notice that there is always such a requirement since at each stage almost all *Q*-requirements are initialized.) Action:

1. If *R* is initialized, then $R=Q_e^{S}$ for some *e* and $S \in \{U,V\}$. Assume $S=U$: the other case is similar, and is treated by interchanging the roles of *U* and *V*. As in the proof of Theorem 3.2 choose a fresh witness $(x_e^{U}, y_e^{U})$ for *R*, i.e. $(x_e^{U}, y_e^{U})=(2i,2i+1)$ where *i* is bigger than all numbers so far used in the construction, bigger than the length of $r^-_{R,U}$, and $Q_{e}^{U}$ precedes $F_{2i}^{U}$ and $F_{2i+1}^{U}$ in the priority listing of the requirements: by property (*) of the $\Pi ^{-1}_a$-approximation pair $\langle f_e, \gamma _e \rangle$ we may suppose that $f_e(\langle x_e^U, y_e^U \rangle ,s+1)=1$ but $f^{U}(\langle x_e^U, y_e^U \rangle ,s)=0$, and
  $$\begin{aligned} \gamma _e(\langle x_e^U, y_e^U \rangle ,s+1)= \gamma ^{U}(\langle x_e^U, y_e^U \rangle ,s)=a. \end{aligned}$$
  For each $S \in \{U,V\}$ define the values of $f^S$ and $\gamma ^S$ at $s+1$ to be the same as at *s*; consequently $U[s+1]=U[s]$, and $V[s+1]=V[s]$.
2. Otherwise:
  - $R=P_e^{S}$ for some *e* and $S \in \{U,V\}$, and $P_e$ is still active. Suppose that $S=U$: the other case is similar, and is treated by interchanging the roles of *U* and *V*. Then pick the least (by code) triple $(\sigma , u, v)$ as in the definition of requiring attention; define $r_{R,U}:=\sigma$. On pairs $\langle x,y\rangle$ that are *V*-collapsed following the *V*-collapse of *u* and *v* performed by $P_{e}^{U}$, define $f^{V}(\langle x,y\rangle ,s+1):=1$ and $\gamma ^{V}(\langle x,y\rangle ,s+1):=1$, leaving untouched the other values of $f^V$ and $\gamma ^V$; let also the values of $f^U$ and $\gamma ^U$ at $s+1$ be the same as at *s*. Consequently $U[s+1]=U[s]$ and $V[s+1]=V[s]_{{{\,\mathrm{coll}\,}}{(u,v)}}$.
  - $T=Q_e^{S}$. Assume that $S=U$: the other case is similar, and is treated by interchanging the roles of *U* and *V*. Define $f^{U}(\langle x^{U}_e, y^{U}_e \rangle ,s+1)$ so that $f^{U}(\langle x^{U}_e, y^{U}_e \rangle ,s+1) \ne f_e(\langle x^{U}_e, y^{U}_e \rangle ,s+1)$ and $\gamma ^{U}(\langle x^{U}_e, y^{U}_e\rangle , s+1):=\gamma _e(\langle x^{U}_e, y^{U}_e\rangle , s+1)$, so that we diagonalize *U* against $E_e$ at witness $(x_e^U,y_e^U)$; leave untouched the other values of $f^U$ and $\gamma ^U$, and let the values of $f^V$ and $\gamma ^V$ at $s+1$ be the same as at *s*. Consequently $V[s+1]=V[s]$ and $U[s+1]$ is the equivalence relation such that  if and only if  and coinciding with *U*[*s*] on all other pairs. (As remarked in the verification, no number $x \notin \{x_e^U, y_e^U\}$ lies in the *U*-equivalence classes of $x_e^U, y_e^U$.)

In all cases when we do not redefine $r_{R,S}$, let $r_{R,S}:=r_{R,S}^{-}$, or $r_{R,S}$ is the empty string if *R* is not preceded by $P^{S}$-requirements. At the end of the stage, initialize all *P*- and *Q*-requirements, having lower priority than the requirement that has acted.

*The verification* A standard inductive argument shows that for every requirement *R* there is a least stage $t_{R}$ such that no $R'$ of higher priority than *R* nor *R* itself requires attention or acts at any $s \geqslant t_{R}$. Hence all parameters for *R*, including witnesses $(x_{i}^{S}, y_{i}^{S})$ and the restraints $r_{R,S}, r^-_{R,S}$, for $S \in \{U,V\}$, reach a limit.

An argument similar to Lemma 3.4 enables us to conclude that for every *e*, *s*, if $(x_e^S,y_e^S)$ is still an active witness for $Q^S_e$ at *s*, then at that stage $[x^S_e]_S \cup [y^S_e]_S = \{x_e^S,y_e^S\}$, each equivalence class among $[x_e^S]_S$ and $[y_e^S]_S$ being a singleton if and only if $f_{e}^S(\langle x_e^S,y_e^S \rangle ,s)=1$.

Moreover, by arguments similar to those in the proof of Lemma 3.4, all *U*- and *V*-equivalence classes are finite. Finally, all other requirements *R* are satisfied. Let us check this in the particular case $R=P_e^U$, leaving the other cases to the reader. Let $t'_R$ be a stage after which no requirement $R'$ having higher priority than *R* requires attention and the finite amount of restraint, imposed (including the strings $r^-_{R_U}$ and $r^-_{R_V}$) imposed by higher priority requirements, does not change any longer. Suppose now that $W_e^{U}$ is infinite. Then by the finiteness of $r^-_{R,U}$, $r^-_{R,V}$, and of the finitely many equivalence classes restrained by the higher priority requirements, after $t'_{R}$ $W_e^{U}$ certainly enumerates a pair of distinct *u*, *v* of the same parity which can be safely *V*-collapsed. Then after $t'_{R}$ eventually there are $\sigma , u,v$ which are eligible to make $P_{e}$ require attention if $P_e$ is still active. In this case, when this happens, the action required by the construction restrains $u,v \in W_{e}^{U}$ and permanently makes , achieving that $W_{e}^{U}$ is not a transversal for *V*.

Finally, by an argument similar to the one employed in the proof of Theorem 3.2 one can also conclude that $U,V \in \Sigma ^{-1}_{a}$, as the pairs $\langle f^{U}, \gamma ^{U}\rangle$ and $\langle f^{V}, \gamma ^{V}\rangle$ are $\Sigma ^{-1}_{a}$-approximation pairs to *U*, *V*, respectively. With respect to Theorem 3.2, there is indeed the additional restraining activity required by *P*-requirements to be accounted for, when at a stage $s+1$ we define $r_{R,S}=\sigma$ for a suitable $\sigma$, but since $\sigma \subset S[s]$ this action does not introduce any changes in $\gamma ^S(\_,s+1)$.

To finish off the proof, the case when ${\mathcal {X}}=\Pi ^{-1}_a$ for some notation *a* with $|a|_{{\mathcal {O}}}>1$ is treated exactly as in Theorem 3.2. $\square$

As anticipated by Remark 3.3, Theorem 3.2 immediately follows from Theorem 4.6 and Proposition 4.3.

Our goal now is to prove that no pair of mutually dark equivalence relations can have infimum. To show this, we make use of the operation $R \mapsto R \oplus {{\,\mathrm{Id}\,}}_1$, that has been greatly exploited in [3]. This operation can be viewed as an inverse of the operation on equivalence relations obtained by collapsing two equivalence classes, and leading from an equivalence relation *R* to $R_{{{\,\mathrm{coll}\,}}(x,y)}$. In fact, if *R* is an equivalence relation and *z* is a number such that $[z]_R$ is not a singleton, then define $R_{[z]}$ to be the equivalence relationSo we see that in getting $R_{[z]}$ instead of collapsing two equivalence classes we do exactly the opposite, i.e. splitting an equivalence class into two classes. We have:

### Lemma 4.7

For every *z* such that $[z]_R$ is not a singleton, $R\oplus {{\,\mathrm{Id}\,}}_1 \equiv _c R_{[z]}$.

### Proof

To show $R_{[z]} \leqslant _c R\oplus {{\,\mathrm{Id}\,}}_1$, consider the computable function *f* where

$$\begin{aligned} f(x)= {\left\{ \begin{array}{ll} 1, &{}\text {if } x=z,\\ 2x,&{}\text {if } x \ne z. \end{array}\right. } \end{aligned}$$

To show $R\oplus {{\,\mathrm{Id}\,}}_1 \leqslant _c R_{[z]}$, pick $y\ne z$ in the equivalence class of *z*, and consider the computable function *g*,

$$\begin{aligned} g(x)= {\left\{ \begin{array}{ll} \frac{x}{2}, &{}\text {if } x \text { even and } \frac{x}{2} \ne z,\\ y, &{}\text {if } x \text { even and } \frac{x}{2}= z,\\ z,&{}\text {if } x \text { is odd}. \end{array}\right. } \end{aligned}$$

$$\square $$

The following lemma has been proved in [3] for ceers (see Observation 4.2 and Lemma 4.6 of [3]), but the same proof works whatever equivalence relation *R* one starts with.

### Lemma 4.8

If *R* is dark then $R<_c R \oplus {{\,\mathrm{Id}\,}}_1$.

### Proof

We recall how the proof goes. First of all, one shows that if *R* is dark then *R* is *self-full* i.e. any reduction $f:R\leqslant _c R$ must have range that intersects all equivalence classes: indeed, if *f* were a reduction missing, say the equivalence class of *a*, then the orbit ${{\,\mathrm{orb}\,}}_f(a)$ would be easily seen to provide a transversal for *R*. Next, one easily shows that if *S* is a self-full equivalence relation then $S<_c S\oplus {{\,\mathrm{Id}\,}}_1$ (in fact it is shown in [3] that *S* is self-full if and only if $S<_c S\oplus {{\,\mathrm{Id}\,}}_1$). $\square$

### Lemma 4.9

If $R,S \in \Sigma ^{-1}_a$ and $R |_d S$ then *R*, *S* do not have $\inf$ in the $\Sigma ^{-1}_a$ equivalence relations.

### Proof

Let $R,S \in \Sigma ^{-1}_a$ be such that $R |_dS$, and suppose that *T* is an infimum of *R*, *S* in $\Sigma ^{-1}_a$, i.e. $T \leqslant _c R,S$ and for every *Z* such that $Z\leqslant _c R,S$ we have $Z \leqslant _c T$. It is clear that *T* is not finite. Let *f*, *g* be computable functions reducing *T* to *R* and *S*. We claim that *f* and *g* do not hit respectively all the *R*-classes and all the *S*-classes, i.e. there exist numbers $y_{R}, y_{S}$ such that for every *x*,  and , respectively. Suppose for instance that for every *y* there exists *x* such that . Then it is easy to define an *R*-computable function $f^{*}$ reducing $R\leqslant _c T$: just set $f^{*}(y)=x$ where *x* is the least number such that . It follows that $g\circ f^{*}$ is an *R*-computable function reducing $R\leqslant _{\deg _{T}(R)}S$ contradicting that $R|_{d}S$. In a similar way one shows that the range of *g* avoids some *S*-classes.

We now derive a contradiction by showing that $T\oplus {{\,\mathrm{Id}\,}}_1 \leqslant _c R,S$ and applying Lemma 4.8. We use the existence of $y_{R}$ to show that a suitable reducing computable function $f^{-}$ reducing $T\oplus {{\,\mathrm{Id}\,}}_1$ to *R* is given by

$$\begin{aligned} f^{-}(x)= {\left\{ \begin{array}{ll} f(x), &{}\text {if } x \text { even},\\ y_{R}, &{}\text {if } x \text { odd}. \end{array}\right. } \end{aligned}$$

A similar argument shows that $T \oplus {{\,\mathrm{Id}\,}}_1 \leqslant _c S$. $\square$

We are now in a position to prove.

### Theorem 4.10

For $a\in {\mathcal {O}}$ such that $|a|_{{\mathcal {O}}}>0$, there are two properly $\Sigma ^{-1}_{a}$ equivalence relations without infimum.

### Proof

By Theorem 4.6 and Lemma 4.5 let *R*, *S* be two equivalence relations lying properly in $\Sigma ^{-1}_{a}$ such that $R|_{d}S$. Then by Lemma 4.9*R* and *S* have no $\inf$. $\square$

Having shown that no pair of mutually dark *c*-degrees can have infimum, it is natural to ask whether one can obtain the same with dark equivalence relations. The next result answers negatively this question: there are infinitely many levels of the Ershov hierarchy which properly contain a pair of *c*-incomparable dark equivalence relations with infimum. This contrasts to the case of ceers (where no pair of dark ceers have infimum, see [3]) and thus vindicates the idea that mutual darkness is the correct analogous of darkness for $\Delta ^0_2$ equivalence relations.

### Theorem 4.11

1. There are *c*-incomparable dark equivalence relations $R,S \in \Pi ^{-1}_2$ such that *R* and *S* have an infimum.
2. For every $a\in {\mathcal {O}}$ such that $|a|_{{\mathcal {O}}}>0$, there are dark $\Delta ^0_2$ equivalence relations $E,F\notin \Sigma ^{-1}_a$ such that *E* and *F* have an infimum.

### Proof

(1) Let $({\mathbf {x}}_m,{\mathbf {y}}_m)$ be a minimal pair of c.e. *m*-degrees. We choose c.e. sets $X\in {\mathbf {x}}_m$ and $Y\in {\mathbf {y}}_m$. We also choose a dark ceer *Q*.

We will show that the equivalence relations $R := F_X\oplus Q$ and $S := F_Y\oplus Q$ have infimum $T = {{\,\mathrm{Id}\,}}_2\oplus Q$ (recall that by $F_X$ we denote the equivalence relation consisting of exactly two classes: *X* and ${\overline{X}}$). It is clear that *T* is a lower bound for *R* and *S*, and *R* and *S* are $\Pi ^{-1}_2$ relations. In addition, the darkness of *Q* ensures that *R* and *S* are dark.

Assume that *E* is a lower bound of *R* and *S*. Consider a reduction $g:E \leqslant _c R$. Then exactly one of the following three cases holds:

- ${{\,\mathrm{range}\,}}(g)$ does not contain even numbers. Then the function $g_1:x \mapsto [g(x)/2]$ is a reduction from *E* to *Q*, and we have $E\leqslant _c Q \leqslant _c T$.
- There is only one class $[w]_E$ such that $g([w]_E)\subseteq 2\omega$. Then the set $[w]_E$ is computable, and the function  gives a reduction from *E* to ${{\,\mathrm{Id}\,}}_1\oplus Q$. Thus, $E\leqslant _c {{\,\mathrm{Id}\,}}_1 \oplus Q \leqslant _c T$.
- There are two different classes $[u]_E$ and $[v]_E$ such that $g([u]_E\cup [v]_E)\subseteq 2\omega$. Note that if  and , then *g*(*x*) must be an odd number.
  We distinguish two cases. If the class $[u]_E$ is computable, then it is easy to show that $E\leqslant _c {{\,\mathrm{Id}\,}}_2\oplus Q$. Assume that $[u]_E$ is non-computable. Without loss of generality, suppose that $[u]_E$ is a co-c.e. set and $[v]_E$ is c.e. Hence, $[u]_E\leqslant _m {\overline{X}}$. Recall that $E\leqslant _c S$, and the relation *S* contains only one non-computable co-c.e. class, namely, the *S*-class $\{ 2z \,:z\in {\overline{Y}}\}$. Thus, we deduce that $[u]_E \leqslant _m {\overline{Y}}$. Hence, the choice of the sets *X* and *Y* implies that the set $[u]_E$ must be computable (as its complement would be *m*-reducible to both *X* and *Y*, and thus would be computable), which gives a contradiction.

In each of the cases above, we showed that $E\leqslant _c T$, therefore, *T* is the greatest lower bound of *R* and *S*.

(2) The proof of the second part is similar to the first one, modulo the following key modification: One needs to choose $\Delta ^0_2$ sets *X* and *Y* such that $X,Y \not \in \Sigma ^{-1}_a$ and the *m*-degrees $\deg _m(X)$ and $\deg _m(Y)$ form a minimal pair. The existence of such sets is guaranteed by the following more general theorem: we prove that, in any $\Sigma$-level of the Ershov hierarchy that corresponds to a successor ordinal (i.e., having notation $2^{a}$ for some *a*), there are *T*-degrees that form a minimal pair and do not contain any set of a lower $\Sigma$-level. Note also that any minimal pair with respect to $\leqslant _T$ is also a minimal pair with respect to $\leqslant _m$.

### Theorem 4.12

For every notation $a\in {\mathcal {O}}$, there are $\Sigma ^{-1}_{2^a}$ sets *X*, *Y* such that $\deg _T(X)$ and $\deg _T(Y)$ form a minimal pair and do not contain $\Sigma ^{-1}_a$ sets.

### Proof

This theorem is a combination of the two following results. The first one is Selivanov’s result [24] about properness of every level in the Ershov hierarchy relative to Turing reducibility. The second one is Yates’ construction of a minimal pair of c.e. Turing degrees (see, e.g., [25]). Below we sketch how these two constructions can be combined together.

We satisfy the following infinite sequence of requirements (recall that, by Posner’s trick, we can consider the same index in an *N*-requirement, see [25]):

$$\begin{aligned}&N_e: \Phi _e^X = \Phi _e^Y = f \ \ \text { is total} \Rightarrow f \ \ \text {is computable},\\&Q^X_e: X \ne \Psi _e^{E_e} \vee E_e \ne \Theta _e^X,\\&Q^Y_e: Y \ne \Psi _e^{E_e} \vee E_e \ne \Theta _e^Y, \end{aligned}$$

where $\{ \Phi _e \}_{e \in \omega }$ is an effective list of all Turing functionals, and $\{ \Psi _e, \Theta _e, E_e \}_{e \in \omega }$ is an effective list of all possible triples consisting of a pair of Turing functionals and a $\Sigma ^{-1}_a$ set. As before, we consider $\langle f_{e}, \gamma _{e}\rangle$ as a $\Sigma ^{-1}_a$-approximation pair to $E_{e}$. We also build pairs $\langle f^X, \gamma ^X \rangle$ and $\langle f^Y, \gamma ^Y \rangle$ in order to get *X* and *Y*.

*Strategies for the requirements and their interactions* In the following we freely adopt language and terminology (length-agreement functions, tree of strategies, use functions, etc.: in particular a small Greek letter denotes the use function of a Turing functional denoted by the corresponding capital Greek letter) which belong to the jargon of the (infinite) priority method of proof: for details the reader is referred to [25].

A *Q*-requirement in isolation can be satisfied using Cooper’s idea in [6], as adapted by Selivanov [24] to the infinite levels of the Ershov hierarchy. Without loss of generality, we consider the strategy for $Q^X_e$, also we assume that each functional is nondecreasing by stage and increasing by argument. For the sake of convenience consider the following length-agreement function:

$$\begin{aligned} l(X,e,s)= & {} \mu z ( \forall x \leqslant z \ \ (X(x)[s] \\= & {} \Psi _e^{E_e}(x)[s] \wedge \Theta ^X_e \upharpoonright \psi _e(x)[s] = E_e \upharpoonright \psi _e(x)[s])). \end{aligned}$$

Thus the strategy for $Q^X_e$ works as follows:

1. Choose a fresh witness $$x_e = x $$ at stage $$s_0$$, thus $$f^X(x,s_0) =0$$ and $$\gamma ^X(x,s_0) =2^a$$.
2. Wait for a stage $s_1 > s_0$ such that $x \leqslant l(X,e,s_1)$.
3. “Put” *x* into *X*, namely define $f^X(x,s_1+1) :=1$ and $\gamma ^X(x,s_1+1) :=a$.
4. Wait for a stage $s_2 > s_1$ such that $x \leqslant l(X,e,s_2)$.
5. Thus at stage $s_2$ we have that $f_e(z,s_1) \ne f_e(z,s_2)$ and $a \geqslant _{\mathcal {O}} \gamma _e(z,s_1) >_{\mathcal {O}} \gamma _e(z,s_2)$ for some element $z < \psi _e(x)[s_1]$. Moreover, from this stage on we have (in case *X* changes only at *x*) that if $x \leqslant l(X,e,s)$ then $f^X(x,s) =0$ if and only if $f_e(z,s) = f_e(z,s_1)$. This means that by putting and extracting *x* we force *z* to go in and out from $E_e$.
6. Thus, we define $f^X(x,s_2+1) :=0$ and $\gamma ^X(x,s_2+1) := \gamma _e(z,s_2)$.
7. At later stages *s*, if we see that $$x \leqslant l(X,e,s)$$ then define $$f^X(x,s+1) := 1 - f^X(x,s) $$ and $$\gamma ^X(x,s+1) := \gamma _e(z,s)$$. Clearly, if later $$f_e(x,t) \ne f_e(x,s)$$ for some $$t>s$$ then $$\gamma _e(z,t) <_{\mathcal {O}} \gamma _e(z,s)$$. Thus, at stage *t*, if $$x \leqslant l(X,e,t)$$ then we can act the same as at stage *s* and define (in particular) $$\gamma ^X(x,t+1) = \gamma _e(z,t) <_{\mathcal {O}} \gamma ^X(x,s+1)$$.

Notice:

- If $Q^X_e$ can keep *X* restrained below $\theta _e(\psi _e(x))$ then it is enough for the win.
- The function $\gamma ^X(x,s)$ always has the possibility to be defined as notation of a smaller ordinal unless $\gamma _e(z,s) = 1$ (note that when $\gamma _e(z,s)$ turns into 1 the function $\gamma ^X(x,s)$ has the possibility to have a last change, thus after this change for any $t > s$ we never see $x < l(X,e,t)$ and the strategy becomes satisfied).

Therefore, each $Q^X_e$-strategy changes *X*(*x*) finitely many times and wins (if some initial part of *X* is restrained). The restraint can easily be achieved by initialization of lower priority strategies. The strategy for $Q^Y_e$ works in the same way. Thus, each *Q*-strategy is a finitary strategy and has only one outcome *fin* on the tree of strategies.

An *N*-requirement in isolation is satisfied by waiting for an expansionary stages and restraining either $\Phi _e^X$ or $\Phi _e^Y$. The definition of an *e*-expansionary stage is as usual. Namely, let the length-agreement function be defined as follows:

$$\begin{aligned} l(e,s) = \mu z ( \forall u \leqslant z\ \ \Phi _e^{X}(u)[s]\downarrow = \Phi _e^{Y}(u)[s]\downarrow ), \end{aligned}$$

and define a stage *s* to be *e-expansionary* if $l(e,s) > l(e,t)$ for all $t < s$ (we also assume that 0 is expansionary). The goal of a strategy $N_e$ is to build a computable function: thus for a given *u* it waits for the first *e*-expansionary stage which covers *u*. At this stage *s* the values of $\Phi _e^{X}(u)[s]\downarrow$ and $\Phi _e^{Y}(u)[s]$ are the same as $\Phi _e^{X}(u)$ and $\Phi _e^{Y}(u)$ (unless *N* is initialized). Thus, each *N*-strategy has two outcomes $\infty < fin$ on the tree of strategies, where it is satisfied vacuously below outcome *fin* and build a computable function below outcome $\infty$.

The tree of strategy is a subtree of $\{ \infty< fin \}^{< \omega }$ with the usual ordering of nodes. At level $k=2e$ we put copies of the strategy $N_e$, at level $k=4e+1$ we put copies of $Q^X_e$, and at level $k=4e+3$ we put copies of $Q^Y_e$.

Similar to the construction of a minimal pair we analyze the most problematic case of interaction between strategies, namely the work of an *N*-strategy with several *Q*-strategies below its infinite outcome. So, let $\eta ^{\smallfrown }\infty \subset \alpha _1 \subset \alpha _2 \subset \cdots \subset \alpha _k$. Assume that each *Q*-strategy $\alpha _i$ has current witness $x_i$. Then it holds that $x_i< \theta _i(\psi _i(x_i))< x_{i+1} < \theta _{i+1}(\psi _{i+1}(x_{i+1}))$ for $1 \leqslant i < k$. Now, if $\alpha _i$ acts at stage *s* [in particular, either $f^X(x_i,s)$ or $f^Y(x_i,s)$ is changed] then $x_{i+1}$ and all greater witnesses are canceled. Also we can visit any of these nodes $\alpha _j$, where $j<i$, only when we get the next $\eta$-expansionary stage $t>s$, which means that $\Phi ^X_e(u)[s] \downarrow = \Phi ^Y_e(u)[s] \downarrow = \Phi ^Y_e(u)[t] \downarrow = \Phi ^X_e(u)[t] \downarrow$ for any $u < l(e,s)$. Therefore, if some $\alpha _j$, where $j<i$, acts at stage *s* then $\eta$ continues to win, moreover $\alpha _i$ is initialized and $\alpha _j$ continues to win too since $\theta _j(\psi _j(x_j))$ was smaller than $x_i$ (note also that it does not matter whether $\alpha _j$ works with *X*- or *Y*-side).

Below we sketch construction and verification, which can easily be expanded to more formal versions.

*The construction* At stage $s+1$ we construct a computable approximation (with the help of substages) of the true path on the tree of strategies. Starting from the root node we perform actions relative to the visited node and decide its outcome, then we visit the node below the outcome and continue until we reach the node of length *s*. Proceeding to the next stage, we initialize all nodes to the right to, or below, the visited one.

If we work with an *N*-strategy $\eta$ at substage $t+1$ then we check whether the stage $s+1$ is $\eta$-expansionary. If it is so then $\eta$ has outcome $\infty$, otherwise $\eta$ has outcome *fin*.

If we work with a $Q^X$-strategy (the case of a $Q^Y$-strategy is totally similar) $\alpha$ at substage $t+1$ then we assign a fresh witness $x_{\alpha } = x$ (bigger then any number mentioned so far) and initialize all nodes below $\alpha$. If witness *x* was already assigned then check whether $x \leqslant l(X,\alpha ,s)$. If the answer is “no” then just take outcome *fin*; if the answer is “yes” then initialize all nodes below $\alpha$ and also define $f^X(x,s+1) :=1$ and $\gamma ^X(x,s+1) :=a$; moreover if the answer “yes” already happened at least one time (after assigning *x*) then there is the least *z* such that $f_{\alpha }(z,s_1) \ne f_{\alpha }(z,s_0)$ and $\gamma _{\alpha }(z,s_1) <_{\mathcal {O}} \gamma _{\alpha }(z,s_0) \leqslant _{{\mathcal {O}}} a$, where $s_0+1$ is a stage such that $\gamma ^X(x,s_0+1) =a$ and $\gamma ^X(x,s_0) =2^a$ (namely, it is the stage when we initiated the attack using *x*) and $s_1+1$ is the next stage with answer “yes”, then define $f^X(x,s+1) :=1 - f^X(x,s)$ and $\gamma ^X(x,s+1) := \gamma _{\alpha }(z,s)$, and initialize all strategies below $\alpha$. In all of the cases the outcome is *fin*.

*The verification* The true path *TP*, defined as the leftmost path on the tree of strategies visited infinitely often, clearly exists. It remains to show that each requirement is satisfied by a strategy on the true path. However, most of the arguments have been already presented in the previous discussions. Also, by induction it is easy to see that each strategy is initialized and initializes other strategies only finitely many times.

If $\eta \in TP$ is an *N*-strategy then it clearly satisfies the corresponding requirement. Namely, waiting for $\eta$-expansionary stages allows to correctly see the value of the computation (also we assume that it happens after stage $s_0$ after which $\eta$ is not initialized). If $\alpha \in TP$ is a $Q^X$-strategy, then let $s_0$ be a stage after which $\alpha$ is not initialized. Henceforth, after assigning the witness *x* the strategy $\alpha$ either sees $x > l(X,\alpha ,s)$, or several times it sees $x \leqslant l(X,\alpha ,s)$ [which immediately forces $f^X(x,s)$ to be changed by the construction]. As previously argued, the case $x \leqslant l(X,\alpha ,s)$ can happen only finitely many times [we always have $\gamma _{\alpha }(z,s) < \gamma ^X(x,s)$ which allows at least one more change for $\gamma ^X(x,s)$]. $\square$

Going back to the proof of item (2) of Theorem 4.11, we can now argue as in (1) by using the sets *X*, *Y* constructed in the previous theorem. $\square$

## The problem of the existence of suprema

In this section we consider the problem of when a given pair of $\Delta ^0_2$ equivalence relations has a supremum. The problem is somehow more delicate than the one concerning infima, discussed in the previous section. This is because a priori the existence of a supremum depends on the level of the Ershov hierarchy that we choose to consider: E.g., consider $R,S\in \Sigma ^{-1}_a$ and $a<_{{\mathcal {O}}} b$. Since $R\oplus S \in \Sigma ^{-1}_a$ is an upper bound of $\{ R,S\}$, if *T* is a supremum of *R* and *S* at the $\Sigma ^{-1}_b$ level, then $T\in \Sigma ^{-1}_a$ as well and *T* is the supremum at the $\Sigma ^{-1}_a$ level. On the other hand, *R* and *S* can have $\sup$ inside $\Sigma ^{-1}_a$, but not have $\sup$ inside $\Sigma ^{-1}_b$. Therefore, we split the problem in two parts: we first provide a necessary condition for the nonexistence of suprema in $\Delta ^0_2$, and then we restrict the focus to the $\Sigma ^{-1}_a$ equivalence relations.

### Nonexistence of suprema in $\Delta ^0_2$

The next theorem shows that mutual darkness forbids the existence of suprema in $\Delta ^0_2$.

#### Theorem 5.1

If $R,S\in \Delta ^0_2$ are mutually dark, then they have no $\sup$ in $\Delta ^0_2$.

#### Proof

Towards a contradiction, assume that $T\in \Delta ^0_2$ is the supremum of *R*, *S*. We build $U \in \Delta ^0_2$ such that *U* is an upper bound of *R*, *S* and avoids the upper cone of *T*. That is to say, we build *U* satisfying the following requirements

$$\begin{aligned}&P_e: \varphi _e \text { does not reduce } T \text { to } U,\\&Q^R: R \leqslant _c U,\\&Q^S: S \leqslant _c U. \end{aligned}$$

*Strategy for the requirements* Call the *R-part* (resp. *S-part*) of $R\oplus S$ the one consisting of all even (odd) numbers. Our idea is to construct $U\supseteq R \oplus S$ by injectively merging equivalence classes of the *S*-part of $R \oplus S$ with classes of *R*-part of $R \oplus S$ (and by these actions meeting the above requirements). Eventually, each equivalence class of *U* will consist of the union of exactly two equivalence classes of $R \oplus S$, one belonging to its *R*-part and the other to its *S*-part.

The strategy for meeting a single $P_e$-requirement is straightforward. We look for a pair of distinct numbers *u*, *v* such that $\varphi _e(u)\downarrow =x_e,\varphi _e(v)\downarrow =y_e$ and $x_e, y_e$ have different parity. When found, we distinguish two cases:

1. If , we do nothing and keep $[x_e]_U$ and $[y_e]_U$ separate;
2. If , we merge $[x_e]_U$ and $[y_e]_U$ and keep them together.

The strategy prevents $\varphi _e$ from being a reduction of *T* to *U*. Indeed, if $\varphi _e$ is total and fails to converge on elements with different parity, then ${{\,\mathrm{range}\,}}(\varphi _e)$ must be all contained in either the *R*-part or the *S*-part of $R\oplus S$. Without loss of generality assume ${{\,\mathrm{range}\,}}(\varphi _e)$ is a subset of the *S*-part of $R\oplus S$. If so, one can easily construct from $\varphi _e$ a computable *f* reducing *T* to *S*. But since $R\leqslant _c T$, we would have $R\leqslant _c S$, contradicting the fact that *R* and *S* are incomparable.

To deal with *Q*-requirements, we let *U* be initially equal to $R \oplus S$. So at first stage of the construction we have that $R\leqslant _c U$ via $f(x)=2x$ and $S \leqslant _c U$ via $g(x)=2x+1$. We claim that this is not injured by the subsequent action of any *P*-requirement. To see why, consider for instance *R*. *R* is obviously reducible to $R \oplus S$. Now let *U* be obtained from $R \oplus S$ by merging a class of its *R*-part (say, $[2k]_{R \oplus S}$) with a class of its *S*-part (say, $[2j+1]_{R \oplus S}$). Then $R\leqslant _c U$, since no equivalence classes in the range of $f(x)=2x$ have been collapsed. A similar reasoning can be made for *S*. Hence if we allow $P_e$ to merge only equivalence classes with different parity, we obtain that the *Q*-requirements are automatically satisfied.

*Interaction between strategies* To combine all strategies, we have to address the following difficulty. When $P_e$ wants to collapse a given pair of equivalence classes we need to be careful that this does not imply collapsing also (by transitivity) equivalence classes of the same parity, because if this happens we might injure a *Q*-requirement. Suppose for instance that $P_i$ wants to collapse $[2k]_U$ and $[2j+1]_U$ but $P_e$ already collapsed $[2k]_U$ and $[2i+1]_U$. If we let $P_i$ be free to act, then by transitivity it would collapse two equivalence classes of the same parity, i.e., $[2j+1]_U$ and $[2i+1]_U$. To avoid this, we define the following priority ordering of the requirements

$$\begin{aligned} P_0< P_1< \cdots P_e < \cdots \end{aligned}$$

Next, $P_e$ looks for numbers *u*, *v* such that $\varphi _e(u)$ and $\varphi _e(v)$ have different parity and come from *fresh* equivalence classes of $R\oplus S$, i.e., neither *u* nor *v* belongs to equivalence classes already collapsed in the construction. A similar condition seems $\Sigma ^0_2$: $P_e$ asks whether there exist *u*, *v* not being in the union of finitely many $\Delta ^0_2$ sets (i.e., the equivalence classes already collapsed). Nonetheless, by making use of the fact that *R* and *S* are mutually dark we will prove that ${\mathbf {0}}'$ can decide such condition (see Lemma 5.2).

*The construction* We build *U* in stages, i.e., $U=\bigcup _{k\in \omega } U[k]$. During the construction we keep track of the equivalence classes that we collapse by putting a witness them in a set *Z*.

*Stage* 0 $$U[0]:=R\oplus S$$ and $$Z:=\emptyset $$.

*Stage* $s+1=2e$ We deal with $P_e$. In doing so, we execute the following algorithm (which is computable in ${\mathbf {0}}'$): List all pairs of distinct numbers (*u*, *v*) until one of the following cases happens

1. $$\varphi _e(u)\downarrow =x_e,$$ $$\varphi _e(v)\downarrow =y_e$$, and
  - either ,
  - or $x_e$ and $y_e$ have different parity and $\lbrace x_e,y_e \rbrace \cap Z=\emptyset$;
2. there is $$\varphi _e(x)\uparrow $$.

Lemma 5.2 proves that the algorithm always terminate. If it terminates with outcome (1.*b*), let $U[s+1]=U[s]_{{{\,\mathrm{coll}\,}}(x_e,y_e)}$ and put $[x_e]_{R \oplus S}$ and $[y_e]_{R\oplus S}$ in *Z*; if it terminates with outcome (1.*a*) or (2), do nothing.

*Stage* $s+1=2e+1$ We ensure that eventually any *U*-class will be the merging of an *R*-class and a *S*-class. To do so, let $u_{e}$ (resp. $v_{e}$) be the least even (odd) number not in *Z*. Let $U[s+1]=U[s]_{{{\,\mathrm{coll}\,}}(u_e,v_e)}$ and put $[u_e]_{R \oplus S}$ and $[v_e]_{R\oplus S}$ in *Z*.

*The verification* The verification is based on the following lemma.

#### Lemma 5.2

For all $P_e$, the algorithm defined at stage 2*e* terminates.

#### Proof

Assume that there is $P_e$ for which the algorithm does not terminate. This means that $\varphi _e$ is total [otherwise, the algorithm at some point would outcome (2)] and $\varphi _e$ reduces *T* to *U* [otherwise, at some point the algorithm would outcome (1.*a*)]. Moreover, $\varphi _e$ can not hit infinitely many equivalence classes of both the *R*-part and the *S*-part of $R \oplus S$. Otherwise, the algorithm would eventually find a pair of fresh equivalence classes with different parity. Without loss of generality, assume that $\varphi _e$ hits only finitely many classes in the *R*-part of $R \oplus S$. Let $A=\lbrace a_0,\ldots ,a_n \rbrace$ be a set of representatives from such classes and let $B=\lbrace b_0,\ldots ,b_n \rbrace$ be a set of odd numbers such that, for all $0\leqslant i\leqslant n$, . The existence of such *B* is guaranteed by the fact that each equivalence class of the *R*-part of $R\oplus S$ is merged in *U* with an equivalence class of the *S*-part of $R\oplus S$. But then one can define the following $\deg _{T}(R)$-computable reduction from *T* to *U* that hits only the *S*-part of *U*,

$$\begin{aligned} f(x)= {\left\{ \begin{array}{ll} \varphi _e(x) &{} \varphi _e(x) \text { is odd,}\\ b_z, \text{ where } z \text{ is } \text{ such } \text{ that } xRa_z &{}\text {otherwise.} \end{array}\right. } \end{aligned}$$

It follows that $R \leqslant _{\deg _{T}(R)}S$, contradicting the fact that $R|_d S$. $\square$

We are in the position now to show that all *P*-requirements are satisfied. The last lemma guarantees that, given $P_e$, the corresponding strategy terminates with either disproving that $\varphi _e$ is a reduction from *T* to *U* or by providing two equivalence classes that can be collapsed in *U* to diagonalize against $\varphi _e$. The *Q*-requirements are also satisfied because we carefully avoid, within the construction, to collapse classes of the same parity. $\square$

By modifying the last proof, we can obtain something stronger.

#### Theorem 5.3

If $R,S \in \Delta ^{0}_2$ and *R* is *S*-dark or *S* is *R*-dark, then *R*, *S* have no $\sup$ in $\Delta ^0_2$.

#### Proof sketch

Suppose that *S* is *R*-dark. Then the proof of Proposition 4.5 shows that $R\nleqslant _{\deg _T(R)} S$.

Assume that *T* is a $\Delta ^0_2$ equivalence relation, and $T = \sup \{R,S\}$. We construct a $\Delta ^0_2$ equivalence relation *U* by employing precisely the same construction as in Theorem 5.1. In order to verify the construction, it is sufficient to re-prove Lemma 5.2 as follows.

Suppose that for some $e\in \omega$, the algorithm for $P_e$ does not terminate. Then, arguing as above, we may assume that:

1. the function $\varphi _e$ is total,
2. $$\varphi _e:T\leqslant _cU$$, and
3. $\varphi _e$ does not hit infinitely many classes of either the *R*-part or the *S*-part of the relation $R\oplus S$.

Thus, one of the following two cases holds.

*Case 1.* The function $\varphi _e$ hits only finitely many classes in the *R*-part and infinitely many classes in the *S*-part. Then the same argument as in the proof of Lemma 5.2 shows that $R\leqslant _{\deg _T(R)} S$, which contradicts the *R*-darkness of *S*.

*Case 2.* Assume that $\varphi _e$ hits infinitely many classes in the *R*-part and only finitely many classes in the *S*-part. Then choose a computable function $h:S\leqslant _cT$, and consider a partial computable function

$$\begin{aligned} f(x) := \left\{ \begin{array}{ll} \varphi _e(h(x))/2, &{}\quad \text {if } \varphi _e(h(x)) \text { is even},\\ \uparrow , &{}\quad \text {otherwise}. \end{array} \right. \end{aligned}$$

Since the function $\varphi _e\circ h$ reduces *S* to *U*, the c.e. set ${{\,\mathrm{range}\,}}(f)$ intersects infinitely many *R*-classes. Therefore, one can choose an infinite *R*-c.e. set *A* with the following properties: $A\subseteq {\mathrm{dom}}(f)$ and  for distinct $x,y\in A$. Note that the condition  implies that , and this, in turn, implies . Hence, *A* is an *R*-c.e. transversal of *S*, which contradicts the *R*-darkness of *S*.

Therefore, one can re-prove Lemma 5.2 and verify the construction. $\square$

The above result contrasts with the fact that ${{\,\mathrm{Id}\,}}$ and a dark ceer have always $\sup$ in the ceers: see [3, Observation 5.1]. Furthermore note that, by reasoning as in Theorem 5.1, one can show that if *R*, *S* are mutually dark equivalence relations of *any* complexity, then they have no sup.

### Nonexistence of suprema at the same level of Ershov hierarchy

We finally turn to the problem of whether there are equivalence relations $R,S \in \Sigma ^{-1}_{a}$ with no supremum in $\Sigma ^{-1}_{a}$. We know from [3] that this is the case for ceers: in particular, there are light ceers with no sup (in the class of ceers). The next proposition extends this fact to all levels of the Ershov hierarchy, with the exception of co-ceers. For what follows, recall that *R* is *essentially* *n-dimensional* if *R* has exactly *n* noncomputable equivalence classes

#### Proposition 5.4

Suppose that ${\mathcal {X}}\in \{ \Sigma ^{-1}_a, \Pi ^{-1}_a \,:|a|_{{\mathcal {O}}} \geqslant 2\}$. There are light equivalence relations *R* and *S* such that both *R* and *S* properly belong to ${\mathcal {X}}$, and *R*, *S* have no $\sup$ in the ${\mathcal {X}}$-equivalence relations.

#### Proof

Fix a set *A* properly belonging to the class ${\mathcal {X}}$. Consider two c.e. sets *U* and *V* such that *U* and *V* are $\leqslant _m$-incomparable. We define the relations *Q*, *R*, and *S* as follows:

$$\begin{aligned} Q := {{\,\mathrm{Id}\,}}\cup \{ (2y,2y+1), (2y+1,2y) \,:y\in A\},\\ R := R_U \oplus Q, \quad S = R_V \oplus Q. \end{aligned}$$

It is easy to show that each of the relations *Q*, *R*, *S* is light and properly belongs to ${\mathcal {X}}$.

Assume that *T* is the supremum of $\{ R, S\}$. Without loss of generality, suppose that $0\in U\cap V$. Since the relation

$$\begin{aligned} E := (R_U\oplus R_V)_{{{\,\mathrm{coll}\,}}(0,1)} \oplus Q \end{aligned}$$

is an upper bound for *R* and *S*, we have $T\leqslant _c E$, and *T* must be essentially 1-dimensional. Let $[a]_T$ be the unique non-computable *T*-class. The conditions $R\leqslant _c T$ and $S\leqslant _c T$ imply that

1 $$\begin{aligned} U\leqslant _m [a]_T \quad \text {and}\quad V \leqslant _m [a]_T. \end{aligned}$$

Consider the essentially 2-dimensional relation $F := (R_U \oplus R_V)\oplus Q$. Since *T* should be reducible to *F*, we have either $[a]_T\leqslant _m U$ or $[a]_T\leqslant _m V$. This fact and (1) together contradict the choice of *U* and *V*. Thus, *R* and *S* have no supremum. $\square$

We conclude the paper with the following open question.

#### Question 1

For which $a \in {\mathcal {O}}$, do there exist equivalence relations properly in $\Sigma ^{-1}_a$ with $\sup$ in $\Sigma ^{-1}_a$?
